# Mechanism and application of yeast and its culture in regulating intestinal antioxidant defense in ruminants

**DOI:** 10.3389/fvets.2025.1657244

**Published:** 2025-08-07

**Authors:** Lan Yang, Xiaoxuan Wu, Dacheng Liu

**Affiliations:** College of Veterinary Medicine, Inner Mongolia Agricultural University, Hohhot, China

**Keywords:** ruminant animals, yeast, oxidative stress, antioxidant, mechanism

## Abstract

In intensive farming mode, oxidative stress is caused by excessive production of reactive oxygen species in ruminants, which seriously threaten animal health and production performance by disrupting intestinal barrier integrity, damaging nutritional metabolism, and inducing inflammatory reactions. Research indicates that yeast supplementation can enhance ruminant health and production performance, while alleviating oxidative stress. Compared to traditional synthetic antioxidants, yeast and its cultures have emerged as preferred solutions due to their multi-target regulatory actions and inherent biosafety. This article focuses on ruminants and integrates recent research findings to systematically review the mechanisms underlying oxidative stress responses in animal organisms, the antioxidant defense system of animals, and the role of yeast and its cultures in enhancing animal antioxidant capacity, to provide ideas for analyzing effective strategies for regulating animal oxidative stress response.

## 1 Introduction

Ruminants constitute a vital component of global agricultural production, where their health status, production performance, and food safety are intrinsically linked to the sustainability of the industry. Under intensive farming systems characterized by high-density rearing and precision nutritional management, environmental stressors exacerbate oxidative damage in these animals (1). Oxidative stress is a cellular damage caused by the accumulation of large amounts of reactive oxygen species, which can lead to dysfunction of the intestinal barrier in ruminants and cause disruption of the gut microbiota, resulting in a significant decrease in nutritional metabolism efficiency and production performance (2). The intestine, as an important organ with both metabolic center and immune barrier functions, enhancing its antioxidant capacity has become an important strategy for improving the health of ruminants.

To alleviate oxidative stress commonly present in ruminant production and ensure animal health, various types of antioxidants have been widely used, including vitamins, trace elements, plant polyphenols, and artificially synthesized antioxidants. Among them, yeast and its metabolites have become a hot research and application topic with their excellent effects.

Yeast and its metabolites have shown significant potential in the field of oxidative stress intervention due to their multi-target regulatory properties. *Saccharomyces cerevisiae* can alleviate damage caused by oxidative stress by directly clearing reactive oxygen species, activating antioxidant enzyme systems (superoxide dismutase, SOD; glutathione peroxidase, GSH-Px, etc.), and regulating gut microbiota (3). The bioactive components such as *β* - glucan and mannan oligosaccharides contained in yeast cultures can not only enhance the antioxidant defense of intestinal epithelium, but also improve cellular stress adaptation by regulating signaling pathways such as Nrf2 and MAPK (4). Research has shown that yeast intervention can simultaneously improve milk production performance and rumen fiber degradation efficiency in dairy cows (5, 6), demonstrating its dual value in metabolic regulation and health maintenance.

Previous reviews have primarily focused on the nutritional regulatory effects of yeast in animals, with inadequate attention given to its antioxidant mechanisms. Current research lacks a systematic elucidation of how yeast synergizes direct and indirect antioxidant actions to regulate the antioxidant system, thereby holistically enhancing organismal antioxidant capacity. This review aims to elucidate these key mechanisms, yielding deeper insights into yeast-mediated antioxidant enhancement.

## 2 Oxidative stress response

### 2.1 Mechanism of occurrence

Oxidative stress is mainly caused by an increase in reactive oxygen species generation and an imbalance in antioxidant defense, leading to the disruption of redox homeostasis. Under physiological conditions, reactive oxygen species is mainly produced by the mitochondrial respiratory chain, nicotinamide adenine dinucleotide phosphate (NADPH) oxidase family, and xanthine oxidase (XO) (7), and participates in cellular signaling. Under pathological conditions, excessive generation of reactive oxygen species or external stimuli (radiation, toxins) can cause dysfunction of the antioxidant enzyme system, leading to damage to the body (8).

The excessive production of reactive oxygen species can lead to an increase in lipid peroxidation products, such as malondialdehyde and 4-hydroxynonenoic acid, which affect cell function by disrupting the integrity of the biofilm structure (9, 10). It also exacerbates cell damage by oxidizing protein thiols and increasing the accumulation of 8-hydroxydeoxyguanosine (11). During this process, excessive reactive oxygen species can activate the NF - κB and MAPK signaling pathways, leading to an increase in pro-inflammatory factors such as tumor necrosis factor - *α* (TNF - α) and interleukin - 6 (IL – 6), thereby forming a vicious cycle between oxidative stress and inflammatory response (12).

### 2.2 Causes of oxidative stress response in ruminant animals

Under intensive farming conditions, oxidative stress is one of the major challenges faced by ruminants. Ruminant animals, due to their unique rumen fermentation system, are more likely to produce metabolites and endotoxins, making the intestinal epithelium more susceptible to oxidative stress. Excessive reactive oxygen species can downregulate the expression of tight junction proteins, weaken intestinal barrier function, and lead to the transfer of endotoxins across the barrier, thereby causing oxidative stress in animal bodies (13). Its occurrence is usually the result of multiple internal and external factors working together. These factors increase the generation of reactive oxygen species or weaken the clearing ability of the body’s antioxidant system, ultimately leading to an imbalance of redox balance. The following explores the causes of oxidative stress in ruminant animals from three aspects: diseases and metabolic disorders, environmental stress, and feed nutritional components.

#### 2.2.1 Diseases and metabolic disorders

The invasion of pathogens into animal bodies triggers an “oxidative burst” of neutrophils and macrophages. Excessive reactive oxygen species can not only kill pathogens, but also directly attack body tissues. For example, in mastitis, reactive oxygen species leads to lipid peroxidation of breast tissue, exacerbating the inflammatory response (14–16). The intestinal barrier damage caused by gastrointestinal infections promotes the translocation of endotoxins (lipopolysaccharide, LPS), activates systemic inflammatory response through the TLR4/NF - *κ* B pathway, triggers the secretion of pro-inflammatory cytokines, and further promotes the generation of reactive oxygen species (17). Parasitic infections induce chronic inflammation by disrupting the integrity of intestinal epithelium, such as nematode disease, significantly increasing serum malondialdehyde (MDA) levels and inhibiting glutathione peroxidase (GPx) activity (18). These sources of infection not only cause local inflammation, but also systemic oxidative damage (19).

Metabolic diseases exacerbate oxidative stress through metabolic imbalance and dysbiosis of the microbiota. Fatty liver is extremely common in high lactation cows. Excessive mobilization of non-esterified fatty acids (NEFA) in fatty liver leads to mitochondrial overload in liver cells, resulting in a large amount of reactive oxygen species leakage from the electron transport chain. At the same time, accompanied by a decrease in antioxidant enzyme activity, lipid peroxidation is induced, causing oxidative stress (20). Under ketosis conditions, the production of *β* - hydroxybutyric acid increases, and its metabolic process is accompanied by a burst of mitochondrial reactive oxygen species, which exacerbates cellular oxidative damage by inhibiting DNA repair enzyme activity (21, 22). Subacute ruminal acidosis (SARA) also causes oxidative stress. High precision feed diets can cause a decrease in rumen pH in ruminants, leading to abnormal proliferation of lactic acid bacteria. At the same time, microbial metabolic disorders can increase the production of reactive oxygen species. Rumen barrier damage promotes the entry of LPS into the bloodstream, activates the TLR4 signaling pathway, and triggers systemic inflammation and oxidative stress (23). It is worth noting that there is a bidirectional interaction between metabolic diseases and oxidative damage. Fatty liver exacerbates endotoxin toxicity by reducing liver detoxification ability, while SARA induced oxidative stress further inhibits rumen epithelial cell proliferation, forming a vicious cycle (24, 25).

#### 2.2.2 Environmental stress

Under high temperature conditions, ruminants rely on respiratory evaporation and skin vasodilation to regulate body temperature, which increases metabolic stress and is accompanied by the generation of reactive oxygen species. At the same time, heat stress caused by abnormal high temperatures can lead to overexpression of heat shock protein 70 (HSP70), which competitively binds to Keap1 protein (26), inhibits nuclear translocation of the Nrf2 signaling pathway, and suppresses the activity of SOD and GPx. Heat stress can also disrupt the balance of rumen microbiota, leading to the proliferation of lactic acid bacteria and a decrease in fiber degrading bacteria, exacerbating rumen acidosis and promoting LPS release. LPS activates the TLR4/MyD88 dependent NF - *κ* B inflammatory pathway, upregulates the expression of pro-inflammatory factors, and forms a vicious cycle of oxidative stress and inflammatory response. Cold stress increases reactive oxygen species generation and inhibits Nrf2 nuclear translocation through mitochondrial uncoupling protein 1 (UCP1) mediated thermogenesis in brown adipose tissue, leading to a decrease in liver antioxidant capacity and accumulation of lipid peroxidation products (27–29).

Long distance transportation, intensive feeding, and early weaning also lead to oxidative damage. Transport stress promotes the secretion of cortisol and catecholamines by activating the hypothalamic pituitary adrenal (HPA) axis and the sensory adrenal medullary (SAM) axis, which generate a large amount of reactive oxygen species (30). Monoamine oxidase (MAO) catalyzes the metabolism of catecholamines while generating hydrogen peroxide, further disrupting the cellular redox homeostasis (31). Under high-density feeding conditions, the increase in temperature and insufficient ventilation in the livestock house promote the accumulation of ammonia (NH_3_) and hydrogen sulfide (H_2_S) concentrations, which directly stimulate the production of reactive oxygen species (32). Early weaning reduces the expression of glutathione synthase (GSS) and gamma glutamylcysteine ligase (GCL) in the intestine, decreases GSH synthesis, weakens the antioxidant barrier function of intestinal epithelium, promotes endotoxin translocation and systemic inflammatory response (33). It may also cause intestinal damage in weaned lambs through the PPAR signaling pathway and iron death, leading to oxidative stress (34).

Heat stress also inflicts significant harm on pregnant ruminants. It elevates the risk of postpartum infections through immunosuppression, impedes mammary gland development, reduces milk production, and induces energy metabolism imbalances that exacerbate the risk of metabolic disorders such as ketosis. Furthermore, maternal heat stress severely compromises fetal intrauterine development by impairing placental angiogenesis and nutrient transport efficiency, resulting in diminished fetal immune competence and substantial health damage (35–38).

#### 2.2.3 Feed nutritional components

Feed, as the main source of energy and nutrition for ruminants, directly determines the health level of the animals in terms of its quality and composition. Excessive grains in high-precision feed diets lead to excessive fermentation of rumen carbohydrates, resulting in the accumulation of volatile fatty acids and a sustained decrease in pH, which promotes the release of LPS by microorganisms (39–41). LPS activates the Toll-like receptor 4 (TLR4) mediated NF - *κ* B inflammatory pathway while inhibiting Nrf2 mediated antioxidant gene expression, systematically reducing GPx activity and promoting the accumulation of lipid peroxidation product malondialdehyde (MDA) (42, 43). The free radicals generated by oxidized fat in spoiled feed can directly attack cell membranes, trigger lipid peroxidation chain reactions, and cause cell apoptosis. At the same time, the interference of spoiled fat on the *β* - oxidation pathway of rumen microorganisms can further exacerbate intestinal oxidative damage (44).

Polluted feed and nutrient deficiencies can exacerbate the risk of oxidative stress. Fungal contaminated feed contains mycotoxins such as aflatoxin and deoxynivalenol, which can induce reactive oxygen species leakage by interfering with the mitochondrial electron transport chain, directly damaging liver and intestinal cells (45, 46). Copper and iron in feed contaminated with heavy metals can catalyze the generation of hydroxyl radicals through the Fenton reaction, leading to oxidative damage to proteins and DNA (47, 48). The lack of nutrients in feed also leads to an imbalance in the antioxidant system. For example, selenium deficiency in feed can reduce the efficiency of GPx enzyme protein synthesis, vitamin E deficiency can weaken the lipid peroxidation defense barrier, and high-precision feed diets can reduce the synthesis of B vitamins by rumen microorganisms, resulting in reduced NADPH regeneration and exacerbating oxidative stress (49, 50).

## 3 Antioxidant system

The animal antioxidant defense system regulates redox homeostasis through a synergistic network of enzymatic and non-enzymatic systems (51). The enzymatic system consists of antioxidant enzymes such as SOD, catalase, and glutathione peroxidase. SOD has three isoenzymes, namely copper zinc superoxide dismutase (Cu/Zn-SOD), manganese superoxide dismutase (Mn-SOD), and extracellular superoxide dismutase (EC-SOD). These three enzymes work together to resist oxidative stress (52). Cu/Zn- SOD and Mn-SOD catalyze the conversion of superoxide anions (O₂^−^) to H₂O₂ and O₂ in the cytoplasm and mitochondria, respectively (53). Catalase (CAT) efficiently decomposes H₂O₂ into H₂O and O₂ in peroxisomes, while GPx relies on reduced glutathione (GSH) to reduce H₂O₂ to H₂O, generating oxidized glutathione (GSSG) that is regenerated by glutathione reductase (GR), forming a dynamic cycle (54, 55).

The non-enzymatic antioxidant system comprises endogenous antioxidants (such as glutathione and melatonin) and exogenous antioxidants (such as vitamin C/E and carotenoids). These molecules collaboratively maintain the body’s oxidative homeostasis by directly neutralizing free radicals and repairing oxidative damage. For example, water-soluble antioxidant vitamin C can directly neutralize reactive oxygen species through single electron transfer; fat soluble antioxidant vitamin E can be embedded in the lipid layer of biological membranes, terminating the chain reaction of lipid peroxidation free radicals (LOO·) through phenolic hydroxyl hydrogenation (56–58), reducing oxidative damage to cell membranes; endogenous polyphenolic metabolites covalently modify Keap1, promote Nrf2 nuclear translocation, activate the expression of antioxidant enzyme genes, and enhance antioxidant capacity (59).

This defense system has a precise regulatory mechanism, where the basal reactive oxygen species level is maintained in a steady state through negative feedback between enzyme activity and substrate concentration. Under stress, Nrf2 mediated antioxidant enzyme synthesis is enhanced, and the repair mechanism triggered by oxidative damage markers such as malondialdehyde and 8-hydroxydeoxyguanosine forms a dual guarantee (60). These two systems jointly create a comprehensive defense network through multi-level regulation, enabling the body to maintain balance under oxidative conditions and prevent cellular oxidative damage.

To enhance this sophisticated defense network, various exogenous substances are often used for nutritional regulation in ruminants. For example, phytochemicals from dietary sources (such as polyphenols) can enhance endogenous enzyme activity by activating the Nrf2 pathway (61, 62); trace elements such as selenium are essential components for key enzyme activity centers such as GPx (63), while certain microorganisms and their metabolites exhibit unique antioxidant regulatory potential (64, 65). Yeast and its cultures, with their unique bioactive components such as *β* - glucan, oligosaccharides, organic selenium, B vitamins, glutathione precursors, etc., can effectively enhance the host’s antioxidant defense network through multi-target and networked regulation, thereby helping to maintain redox homeostasis (66).

## 4 The mechanism by which yeast and its cultures enhance the antioxidant capacity of animal bodies

### 4.1 Characteristics of yeast and its cultures

Yeast, as a group of unicellular fungi widely distributed in nature, is of great value in ruminant nutritional regulation, with *Saccharomyces cerevisiae* and *Candida utilis* being the most widely used. Yeast and their cultures are rich in high-quality proteins, B vitamins, and minerals, and can improve the palatability of feeds (67). In the regulation of the rumen environment, yeast can promote the proliferation of fibrolytic and lactic acid-utilizing bacteria (68) and inhibit the colonization of pathogenic bacteria, thus stabilizing the rumen pH, reducing the risk of subacute acidosis (69–71), and improving the digestibility of dry matter (72, 73). Furthermore, by reducing lactic acid accumulation, yeast can improve milk production efficiency and milk fat percentage (74). In terms of intestinal health, yeast effectively reduces the incidence of diarrhea in young livestock and enhances disease resistance by regulating the structure of the microbiota (39–41, 64, 65, 75), enhancing intestinal barrier function, and activating immune response (76). Of particular importance is its significant antioxidant capacity. Metabolites from yeast, such as glutathione and polyphenols, can enhance the body’s antioxidant capacity (77, 78). By clearing free radicals and increasing antioxidant enzyme activity (such as superoxide dismutase and catalase), the metabolites can alleviate the negative effects of oxidative stress on animals, especially under high temperature or high metabolic load conditions, significantly improving animal health status (79).

### 4.2 Direct antioxidant activity of yeast and its cultures

#### 4.2.1 Glutathione

Yeast enhances the antioxidant capacity of animal intestines by regulating the dynamic defense system centered on GSH. Under oxidative stress conditions, yeast activates a specific transcriptional regulatory network, significantly increasing the expression of gamma glutamylcysteine synthase (*γ* - GCS) and glutathione synthase (GS) (80), enhancing their transmembrane transport capacity, and promoting the synthesis of GSH (81). Yeast breaks down cell walls in the acidic microenvironment of the intestine, delivering the produced GSH to intestinal epithelial cells to maintain cell integrity (82).

GSH exerts its antioxidant function in the intestine through various ways. Its active thiol group (− SH) directly scavenges hydroxyl radicals through electron transfer, interrupting the chain reaction of free radicals. GSH, as a key substrate of glutathione peroxidase, systematically scavenges oxidative products such as H₂O₂ and organic peroxides (ROOH). Reductase systems such as thioredoxin (Trx) and glutaredoxin (Grx) work synergistically with GSH to repair thiol disulfide bond exchange and restore key enzyme activity (83), thereby constructing a multi-level antioxidant barrier (84). Yeast can secrete coenzyme precursors such as riboflavin, which directly enhance glutathione reductase (GR) activity (85), drive the regeneration of oxidized glutathione (GSSG) into reduced glutathione, and maintain intracellular redox homeostasis. In addition, the system extends to the maintenance of intestinal barrier function, protecting tight junction protein structures by clearing reactive oxygen species, promoting the secretion of key components in the mucus layer to form a physical barrier, and regulating the interaction of antioxidant anti-inflammatory signaling pathways (82). Adding yeast and its culture during daily feeding can effectively improve the antioxidant capacity of ruminants. According to the research findings of Chen et al. (86) supplementing with yeast culture increases glutathione levels, effectively enhancing antioxidant capacity.

#### 4.2.2 Superoxide dismutase

Yeast constructs a multi-level antioxidant system by secreting superoxide dismutase (SOD), directly clearing reactive oxygen species and regulating the antioxidant system (87). SOD can catalyze the dismutation of superoxide anions into hydrogen peroxide and oxygen, blocking the oxidative chain reaction of reactive oxygen species. Extracellular Cu/Zn-SOD catalyzes the dismutation reaction of superoxide anions through its copper zinc active center, effectively inhibiting lipid peroxidation and maintaining intestinal mucosal barrier function (39–41, 88), protecting intestinal mucosa from oxidative damage (5, 6). The Mn-SOD targeted by mitochondria efficiently catalyzes O₂^−^ dismutation in the manganese active center, reduces mitochondrial reactive oxygen species accumulation, stabilizes membrane potential, and inhibits abnormal opening of membrane permeability transition pores (mPTP), thereby blocking cytochrome c release and activating apoptosis signals (89). The SOD released after yeast lysis can still maintain its activity in the extracellular environment, continuously clearing reactive oxygen species and forming a dynamic antioxidant defense line.

At the molecular regulatory level, SOD activates the Nrf2 signaling pathway in the body, synthesizes endogenous antioxidant enzymes such as GSH, and inhibits NF - *κ* B-mediated inflammatory responses, blocking the vicious cycle of oxidation inflammation (55, 90). Meanwhile, the H₂O₂ generated by SOD catalysis can be synergistically degraded into water by the host GPx, avoiding oxidative damage caused by the accumulation of H₂O₂ (91).

#### 4.2.3 Vitamins and organic acids

Yeast cultures are rich in vitamins and organic acids, which neutralize reactive oxygen species through multi-level interactions. Vitamin C, as a water-soluble antioxidant, can directly eliminate superoxide anions and hydroxyl radicals in the intestinal lumen (92). Vitamin E (alpha tocopherol) effectively terminates the lipid peroxidation chain reaction by embedding into the phospholipid layer of intestinal epithelial cell membrane, thereby reducing the production of toxic products such as malondialdehyde (MDA) (93, 94). In the B vitamins, riboflavin acts as a cofactor for glutathione reductase (GR), catalyzing the reduction of GSSG to its active form GSH (95, 96). At the same time, tricarboxylic acid (TCA) cycle intermediates such as succinic acid and *α* - ketoglutarate can accelerate the operation of mitochondrial electron transport chains (14–16), generate ATP, and promote the generation of NADPH, thereby enhancing the antioxidant system’s capacity (61, 62, 97, 98).

Adding vitamins and organic acids during daily feeding can effectively alleviate oxidative damage in ruminant animals. According to Wang et al.’s research, supplementing vitamin E and yeast culture effectively reduced the absorption rate of endotoxins in dairy goats, enhanced their antioxidant capacity, and thus alleviated heat stress (99). Supplementing with vitamin E and selenium can effectively improve the physiological, hormonal, and antioxidant status of sheep, and alleviate heat stress (100).

#### 4.2.4 Polyphenols

Yeast metabolizes polyphenol precursors in the diet, such as rutin and chlorogenic acid, and uses extracellular enzymes such as *β* - glucosidase and esterase to hydrolyze them into highly active polyphenol derivatives such as quercetin and caffeic acid (101). This significantly enhances the lipid solubility and bioavailability of polyphenols, and improves the antioxidant capacity of animal intestines (102). Quercetin and other polyphenolic substances efficiently scavenge free radicals through the hydrogen atom transfer (HAT) mechanism of phenolic hydroxyl groups and the single electron transfer (SET) mechanism (29, 103). Caffeic acid, catechins, etc. form highly stable octahedral complexes with Fe^2+^/Cu^2+^ through the phenolic hydroxyl groups in catechol or gallic acid structures, blocking the redox active sites of metal ions, directly blocking the Fenton reaction, and reducing the generation of hydroxyl radicals (104, 105). In addition, hydrophobic polyphenols such as resveratrol and curcumin can be embedded in the membrane of intestinal epithelial cells, directly quenching lipid peroxidation free radicals (LOO·) inside the membrane, thereby maintaining cellular homeostasis (106).

Yeast metabolites can also enhance antioxidant capacity by activating the antioxidant system. For example, quercetin promotes the expression of superoxide dismutase and glutathione peroxidase by activating the Nrf2/KEAP1 pathway (61, 62); caffeic acid can selectively inhibit *Escherichia coli*, while its metabolism of short chain fatty acids (such as butyric acid) promotes the proliferation of lactic acid bacteria and inhibits reactive oxygen species generation (107, 108) (Table 1).

**Table 1:** The impact of yeast culture on its oxidizing ability.

Species	Feeding supplement	Conclusion	References
Cow	*Saccharomyces cerevisiae*	Increase the levels of T-AOC, GSH-Px, and SOD in serum	(66)
Lactating cow	*Chromium yeast*	Enhance the activity of GSH-Px, SOD, and T-AOC in serum, and reduce the concentration of MDA	(143)
Neonatal goats	Mannose-oligosaccharide	Increase serum CAT and IL-4 levels, and decrease MDA and IL-6 levels	(144)
Holstein cows	Se-yeast	Enhance the activity of plasma T-AOC, SOD, and CAT, and maintain the activity of plasma GSH-Px.	(63)
Female holstein calf	Sodium butyrate	Enhance serum GSH-Px activity and reduce serum MDA concentration	(141)
Lamb	*Bacillus licheniformis* and *Saccharomyces cerevisiae*	Enhance serum GSH-Px activity and reduce serum MDA concentration	(145)

### 4.3 Indirect antioxidant effects of yeast and its cultures

#### 4.3.1 Intestinal flora

Yeast enhances the antioxidant capacity of animal intestines by regulating the structure of gut microbiota. Its core mechanism of action is first reflected in the inhibition of pathogenic bacteria. The lectin proteins (such as Flo1p) on the surface of *Saccharomyces cerevisiae* are calcium-dependent glycoproteins that can specifically recognize and adhere to the mannose residues on intestinal epithelial cells. They physically occupy potential colonization sites for pathogenic bacteria (such as *Escherichia coli* and *Salmonella*), forming a biological barrier that competitively inhibits the colonization and proliferation of pathogenic bacteria (109–111). Simultaneously prioritizing the metabolism of carbon sources in the intestine, limiting the energy acquisition and respiratory chain activity of pathogenic bacteria (112). Yeast inhibits the proliferation and toxicity of pathogens by secreting various antibacterial active substances. For example, antimicrobial peptides produced by *Saccharomyces cerevisiae* can target lipopolysaccharides on the surface of *Escherichia coli*, disrupting the integrity of the bacterial outer membrane structure, leading to leakage of intracellular energy substances and significantly weakening bacterial activity (113, 114). These antibacterial mechanisms work together to effectively inhibit the colonization and metabolic activity of pathogenic bacteria, reduce their survival rate, and decrease the release of pro-inflammatory factors such as LPS while reducing the number of pathogenic bacteria. They also inhibit the activation of NADPH oxidase in macrophages, thereby reducing the excessive generation of intestinal reactive oxygen species (115).

Yeast not only inhibits harmful bacterial communities, but also improves the intestinal environment and enriches probiotic communities with antioxidant properties. The mannan and *β* - glucan produced by yeast metabolism can serve as prebiotics, promoting the proliferation of beneficial bacteria such as lactic acid bacteria and *Bifidobacteria* (5, 6, 116), by increasing short-chain fatty acids in the intestine, inhibiting the adhesion and growth of harmful bacteria, and collaborating with the anaerobic microenvironment formed by yeast consuming oxygen, the proliferation of pathogens can be suppressed. The enrichment of these probiotics works together with the anaerobic microenvironment formed by yeast oxygen consumption to inhibit the proliferation of pathogens. Yeast can also increase the abundance of butyrate producing bacteria, which produce short chain fatty acids (SCFAs) by fermenting dietary fiber (24, 25), directly neutralizing reactive oxygen species such as hydroxyl radicals (117–119), and activating the antioxidant defense pathway of intestinal epithelial cells by inhibiting histone deacetylase (HDAC) (120), regulating antioxidant enzyme activity, and directly clearing reactive oxygen species (121). It is worth noting that the lactic acid produced by the metabolism of lactic acid bacteria can lower the pH value of the intestine, inhibit the proliferation of hydrogen sulfide producing bacteria, thereby reducing the production of hydrogen sulfide (H₂S) with strong oxidative toxicity, maintaining intestinal barrier function, and reducing oxidative stress (122, 123).

#### 4.3.2 Intestinal barrier function

Yeast can also regulate intestinal barrier function and resist oxidative stress by strengthening the mucosal barrier and repairing tight connections. Yeast can enhance the mucosal barrier function, and its metabolites can regulate the function of goblet cells through molecular signaling networks. For example, SCFAs and polyphenol derivatives activate the ERK/Sp1 signaling pathway in intestinal epithelial cells, promote the transcription of MUC2 genes in goblet cells, and stimulate the secretion of mucins (124, 125). This significantly increases the thickness and viscosity of the mucus layer, and the thickened mucus layer can effectively block the infiltration of endotoxins and free radicals produced by rumen fermentation into the intestinal epithelium, reducing oxidative stress-induced reactions (57, 58). At the same time, yeast inhibits the activity of sulfatase positive bacteria, reduces the hydrolysis of mucin sulfate groups (126), prolongs the stability of the mucus layer, and thus maintains the stability of the mucus layer (118, 119). This not only enhances the physical barrier ability of the mucus layer, but also reduces the generation of free radical precursors such as sulfides by reshaping the metabolic pattern of the microbiota.

#### 4.3.3 Immunologic function

Yeast cell wall components (such as *β* - glucan) bind to Dectin-1 and TLR2 receptors on the surface of intestinal mucosal macrophages (127, 128), increasing anti-inflammatory factor IL-10 and inhibiting the release of pro-inflammatory factors TNF - *α* and IL-6, thereby blocking NADPH oxidase activation and reducing the production of superoxide anions and hydrogen peroxide (H₂O₂) (9, 10, 21, 22, 129). At the same time, yeast metabolite butyric acid can increase the levels of pro-inflammatory cytokines IL-10 and TGF - β, inhibit Th17 cell activity, reduce IL-17-mediated neutrophil infiltration and myeloperoxidase (MPO) release (130, 131), thereby reducing the production of oxidative toxic substances such as hypochlorous acid (HOCl) (132). In addition, yeast significantly increases the level of secretory IgA by stimulating the differentiation of Peyer’s patches B cells into plasma cells. SIgA specifically binds to pathogen surface antigens, blocking their adhesion to intestinal epithelium and reducing oxidative damage caused by pathogen colonization, thereby alleviating oxidative stress (133, 134). According to research by Du et al. (66) supplementation with brewing yeast culture effectively reduces inflammatory factors in dairy cows and synergizes with endogenous hormones to alleviate the adverse impacts of heat stress. Although Zhang et al. (14–16) observed no significant effect of yeast culture supplementation on immunity in bulls, this discrepancy may be attributable to variations in active components and strains across yeast culture preparations.

Yeast enhances immunity and antioxidant capacity by regulating SCFAs and tryptophan metabolism. The butyric acid produced by the metabolism of butyric acid producing bacteria enters macrophages and T cells through the monocarboxylate transporter (MCT1) (135, 136), inhibiting HDAC activity and activating PPAR *γ*. PPAR γ upregulates the expression of SOD and CAT, while inhibiting key glycolytic enzymes (HK2, PFKFB3) (137), reducing mitochondrial reactive oxygen species leakage (138, 139). Butyric acid can also promote Nrf2 nuclear translocation by inhibiting Keap1, increase the expression of heme oxygenase-1 (HO-1) and glutathione synthase, enhance antioxidant capacity (140–142), and provide dynamic protection for the intestinal health of ruminants.

## 5 Conclusion

During intensive farming of ruminants, intestinal barrier damage, metabolic disorders, and inflammatory reactions caused by oxidative stress severely restrict their health and production performance. This article systematically summarizes the causes of oxidative stress (such as high-precision feed, environmental stress, metabolic diseases) and endogenous antioxidant mechanisms (enzymatic and non-enzymatic system synergy), with a focus on revealing the mechanisms by which yeast and its cultures enhance intestinal antioxidant capacity through multiple dimensions and pathways. On the one hand, by secreting and metabolizing glutathione, superoxide dismutase, and polyphenolic substances, reactive oxygen species is directly cleared and oxidative damage is reduced. On the other hand, by regulating intestinal microbiota and indirectly improving intestinal barrier and immune function, the two work together to enhance intestinal antioxidant defense capacity. Although the application research of yeast in the prevention and control of reactive oxygen species in ruminants is still in the exploratory stage, it provides new ideas for the prevention and control of reactive oxygen species in intensive farming through multi-target antioxidant mechanisms and host microbe synergistic regulation ability. In the future, we can delve deeper into exploring strain specificity, host–microbe interaction mechanisms, and analyzing key yeast metabolites, to promote the precise application of personalized antioxidant solutions in ruminant production.
